# Structural Impediments to Condom Access in a High HIV/STI-Risk Area

**DOI:** 10.1155/2010/630762

**Published:** 2010-08-31

**Authors:** Christine Rizkalla, Laurie J. Bauman, Jeffrey R. Avner

**Affiliations:** ^1^Division of Pediatric Emergency Medicine, Department of Pediatrics, Albert Einstein College of Medicine, The Children's Hospital at Montefiore, Bronx, NY 10467, USA; ^2^Division of Pediatric Emergency Medicine, Maimonides Medical Center in Brooklyn, NY 11219, USA; ^3^Division of General Pediatrics, Department of Pediatrics, Albert Einstein College of Medicine, Children's Hospital at Montefiore, Bronx, NY 10467, USA

## Abstract

As embarrassment is a known obstacle to condom acquisition, selling condoms from physically inaccessible places that require personnel assistance constitutes a barrier to access. This study investigates the extent of this barrier in the Bronx, a high HIV/STI prevalence county of New York. 75 of 320 listed Bronx pharmacies were sampled via computer randomization. Investigators coded condom placement and physical accessibility within these pharmacies and 140 surrounding stores. 91% of sites sold condoms. In 82%, condoms could not be accessed without assistance. Condoms were physically inaccessible in venues most encountered in the community: grocery stores versus pharmacies (OR=15; 95% CI, 5–48), independent versus chain pharmacies (OR=32; 95% CI, 6–235). They were physically inaccessible more in the lowest SES/highest HIV prevalence areas versus the highest SES/lowest HIV prevalence areas (OR = 4.3, 95% CI, 1.1–17). Findings can inform efforts to increase accessibility of condoms, distribute condoms in alternative settings, and prompt similar investigations in other high-risk communities.

## 1. Introduction

New York City has one of the highest reported human immunodeficiency virus (HIV) and acquired immunodeficiency syndrome (AIDS) rates in the United States, more than triple the national average [1]. Approximately one quarter of these HIV-positive individuals live in Bronx County (known as the Bronx), with areas reaching an HIV/AIDS prevalence of greater than 2% [2]. Relative to the United States, New York State, and the other counties in New York City, the Bronx also has a disproportionately higher prevalence of sexually transmitted infections (STIs) and teen pregnancy. In 2005, the teen pregnancy rate in the Bronx was 140% higher than that of New York City, and the Chlamydia case rate was more than double the national rate [3, 4]. Therefore, condom access, as a means of HIV/AIDS, STI, and teen pregnancy prevention, is a high priority in this community. 

Although the determinants of condom use are complex and multifactorial, prior work has established that preparedness by possession of condoms is one strong predictor of use [5–7]. However, it has been well documented that embarrassment during the purchase of condoms remains a barrier to acquisition and use among young adults and women [8–10]. Condoms have been described as a “socially sensitive product”; a real or imagined social presence around such products creates embarrassment, which in turn affects purchasing behavior [8]. By the same token, research has shown that adolescents prefer to purchase condoms from places where they are clearly visible and quickly purchased, even when given a cheaper option [11, 12].

In 2004, Brackett reported that one of several strategies employed by young adults to reduce the embarrassment of condom purchase is to avoid asking for help or for location of condoms within a store [13]. Yet the popular press has reported that condoms are often stored in locked cases in drug store aisles. In 2006, the Washington Post reported that one national pharmacy sold condoms in locked cases in 22 of 50 of their Washington, DC stores [14]. Requiring assistance from store personnel to purchase condoms makes the sale more public, lengthy, complicated, and potentially embarrassing, thereby creating a barrier to access. 

The Theory of Planned Behavior proposes that intentions and perceived control over behavior predict behavioral performance [15]. Part of this model suggests that one's intentions and behavior in performing a given act are related to the control and difficulty one perceives in performing that act. Therefore, among individuals intending to acquire condoms, a setting in which those vulnerable to embarrassment are obliged to ask for assistance creates a structural disincentive with a potentially negative outcome. 

While condom purchase preferences and behavior have been explored, condom placement within physical reach has received narrow attention. In 2006, Scott-Sheldon et al. reported on the impact of embarrassment in securing condoms. They examined condom placement relative to the proximity of other embarrassing products, and the positive/negative associations such placement fosters [16]. In this study, two thirds of condoms were positioned behind or next to the checkout counter, though the proportion behind the counter and requiring assistance is not specified. Klein et al. investigated several aspects of condom availability, including condom visibility and placement within store aisles [12]. This paper found that the majority of drug stores displayed condoms in the aisles, while the majority of private grocery and convenience stores kept them behind the counter. However, the limited data on this barrier have not been validated in a community of such significant risk as the Bronx, nor has condom accessibility through the lens of mandatory interaction been researched. 

The goal of this paper was to describe the prevalence of structural barriers to condom access in the Bronx, specifically physical inaccessibility, defined as the sale from locked cases or behind store counters requiring interaction with store personnel. We hypothesized that physical inaccessibility of condoms would be found in the majority of the sites selling condoms in the Bronx. In addition, we sought to determine whether or not the prevalence of these structural barriers was associated with the socioeconomic status (SES) of the health districts within the Bronx.

## 2. Methods

### 2.1. Study Setting

The Bronx is a densely populated urban community of approximately 1.4 million people, with commercial areas in close proximity to or interspersed within residential neighborhoods. One-third of Bronx residents live below the federal poverty line, though socioeconomic status varies widely between the seven designated health districts of the county. Health data for the Bronx are listed in Table 1 [2, 3]. 

### 2.2. Study Design

We conducted an observational study of grocery stores and pharmacies in the Bronx. The Bronx Yellow Pages lists 320 pharmacies (80 of them are chain pharmacies) and approximately 900 grocery stores in the 20 zip codes in the county. Seventy-five pharmacies were sampled using computerized randomization from the 320 listed. As pharmacies are largely represented in commercial areas, they were used as focus points for sampling of the two nearest grocery stores or supermarkets. If the pharmacies were so closely clustered that two different grocery stores per pharmacy were not encountered, we would survey the maximum number of stores within walking distance of those pharmacies, operationalized as within 5 blocks.

Study representatives were trained by the principle investigator to collect data. Staff entered each selected location in the manner of an ordinary customer and observed the location of condom placement. If condoms were not visible, the representative asked the store clerk whether condoms were sold and if so, where in the store they were located. On leaving the store, the representative provided the following data on a coding form developed by the investigators: the type of store, zip code, availability of condoms, specific condom location within the store, whether personnel assistance was needed to obtain condoms prior to purchase, and the number of interactions required prior to purchase. 

Information regarding SES, HIV, teen pregnancy, and STI prevalence was obtained from the New York City Department of Health (DOH), which provides data for the seven health districts and the 20 zip codes in the Bronx. Each site was assigned to one of these districts based on its zip code, to determine whether the manner of condom sales correlated with the health statistics of that region.

### 2.3. Statistical Analysis

We determined that 75 pharmacies and 90 grocery stores needed to be sampled based on a presumed point estimate of 50% of sites keeping condoms physically out of reach, in order to achieve a 95% confidence interval of 10% around this point estimate. To even the distribution of grocery stores and achieve the minimum sample size, we surveyed the two closest grocery stores to each pharmacy, aiming for 150 stores and 225 total sites. Data organization and analysis were performed using Epi Info version 2000 (EpiInformatics, Doraville, GA). The chi-square test was used to compare categorical data. Ninety-five percent confidence intervals were calculated by standard methods [17]. 

## 3. Results

We sampled a total of 215 sites, 75 pharmacies and 140 stores. Because of the close proximity of some of the pharmacies, two stores per pharmacy were not always encountered for sampling. Condoms were sold at 195 (91%) of these sites (Table 2). Condom purchase required assistance from site personnel at 160 of the 195 sites selling condoms (82%; 95% CI: 76%–87%). The placement of condoms within the sites visited is shown in Table 3. Condoms were more likely to be kept out of reach in the 115 grocery stores (96%) compared to the 75 pharmacies (60%) selling them (OR = 15, 95% CI: 5–48). Condoms were also kept out of reach in more independent pharmacies (78%) compared to chain pharmacies (10%) (OR = 32, 95% CI, 6–235). Four sites required assistance from two or more personnel prior to condom purchase whereas the remainder required assistance from one person prior to purchase. 

We stratified for the SES/HIV prevalence of each Bronx health district relative to the manner of condom distribution. In health districts 1&2, with the greatest HIV, STI, and teen pregnancy prevalence and the greatest number living below the federal poverty level, condoms were kept out of reach in 90% out of 55 sites sampled (Table 4). In health districts 6&7, with the lowest HIV, STI, and teen pregnancy prevalence, and the lowest number living below the federal poverty level, condoms were kept out of reach in 70% out of 30 sites (Table 4). Condoms were more likely to be kept out of reach in the lowest SES/highest infection and teen pregnancy prevalence districts compared to the highest SES/lowest infection and teen prevalence districts (OR = 4.3, 95% CI: 1.1–17).

## 4. Discussion

Although 91% of stores surveyed in the Bronx sold condoms, the vast majority (82%) sold them in locked cases or behind sales counters. In almost all of the convenience stores and in 78% of independent pharmacies, consumers required assistance from site personnel in order to purchase condoms. Thus, condom accessibility was poor in the sites most commonly encountered; most Bronx stores are convenience stores and 3 out of every 4 pharmacies are small and privately owned. Accessibility was likewise poor in the lowest-SES districts. Since the low-SES districts also have the highest rates of HIV, STIs, and teen pregnancy, barriers to condom access are of particular concern. 

The study conducted by Klein et al. in 2001 found that almost two-thirds of adolescents who purchased their condoms did so only from pharmacies [12]. The purchasing preferences reported in Klein's work highlight the potential relevance of our finding that the majority of Bronx pharmacies sold condoms from behind the counter. 

Our findings also differ from those of Scott-Sheldon et al., who report on 66% of condoms being sold from behind the counter or “next to the pharmacist.” While we do not know what proportion of these condoms was sold exclusively from behind the counter, we found that significantly more condoms were sold in this manner, and this practice was more common in a higher-risk community. This reinforces the negative implication of selling the vast majority of condoms in a manner that obligates assistance from a stranger.

A limitation of our study is that condoms can be acquired from sources other than stores, such as high schools or community health centers. There were no data available on the number or rate of condom distribution in local high schools. However, students who have previously reported on condom availability in schools have found them to be inadequate in supply [18]. Moreover, distribution in schools does not benefit those who are older or adolescents who are not in school. A further limitation was inadequate information about where individuals in our region actually acquire their condoms. We were unable to quantify whether or not individuals seek condoms from free sources and the number of such sites in the region. Though we do not know whether young adults usually purchase condoms from chain pharmacies, where condoms are usually sold with open access, those pharmacies represent the minority of the total in our community and may be difficult for some to access. Finally, although the small sample size led to wide confidence intervals around the odds ratios, the results are nonetheless significant as the lower limit of the confidence interval was greater than one. 

Although not confirmed, it has been suggested that the fear of theft is one reason why condoms are sold from locked cases or behind store counters. Therefore, efforts to change the sales practices in smaller stores with less financial stability may be unrealistic. However, this does not change the fact that access to condoms is critical, especially in areas which had the highest STI rates; that there may be a structural barrier to condom purchase in these communities represents an incongruity between what is most needed and what is available. To this end, public health policy must involve local community advocates and businessmen.

## 5. Conclusion

The vast majority of sites surveyed in the Bronx sold condoms in locked cases or behind sales counters. Failure to make condoms readily accessible for unmediated purchase may disproportionately deter adolescents and women from acquiring condoms and ultimately from adopting recommendations for condom use. This information can prompt similar investigations in other high-risk communities and inform public health efforts to increase condom accessibility and the distribution of free condoms in public and practice settings.

## Figures and Tables

**Table 1:** Health statistics for Bronx County.

Health district	% Living below poverty level	HIV diagnosis (per 100,000 individuals)	Pregnancy rates, teens age 15–19 (per 1,000 females)	Gonorrhea case rate (per 100,000 Individuals)	Chlamydia case rate (per 100,000 individuals)
1	45%	143	103	204	791
2	41%	140	96	263	871
3	41%	112	95	217	789
4	33%	91	75	148	612
5	22%	54	60	138	711
6	16%	44	58	134	584
7	15%	28	41	54	242

**Table 2:** Sites surveyed and proportion of condoms requiring assistance to access.

Type of site	*n* (%) selling condoms	*n* (%) requiring assistance prior to purchase	*n* (%) not requiring assistance prior to purchase
Chain pharmacy (*n* = 20)	20 (100%)	2 (10%)	18 (90%)
Independent pharmacy (*n* = 55)	55 (100%)	43 (78%)	12 (22%)
Total pharmacies (*n* = 75)	75 (100%)	45 (60%)	30 (40%)

Convenience store (*n* = 126)	116 (92%)	112 (97%)	4 (3%)
Supermarket (*n* = 14)	4 (29%)	3 (75%)	1 (25%)
Total Stores (*n* = 140)	120 (86%)	115 (96%)	5 (4%)

**Table 3:** Location of condoms within the stores and pharmacies.

Placement of condoms	Frequency (%) (*n* = 195)
Store/pharmacy aisle	22 (11%)
Behind the sales counter	158 (81%)
In front of the counter	11 (6%)
Locked case in aisle	2 (1%)
On the counter	2 (1%)

**Table 4:** Physical accessibility of condoms in the lowest- and the highest-SES districts.

Health district	% of residents living below federal poverty level	% of residents living with HIV/AIDS	Mean pregnancy rates, ages 15–19 (per 1000 females)	Sites with physically inaccessible condoms (%)	Sites with accessible condoms (%)
1, 2	41%–45%	2.3%-2.4%	100	50 (91%)	5 (9%)
6, 7	15%-16%	0.5%–0.8%	50	21 (70%)	9 (30%)

Assistance required to access condoms in lowest-SES districts compared to highest-SES districts: Odds ratio = 4.3 (95% CI, 1.13–17).
